# A Representational Similarity Analysis of the Dynamics of Object Processing Using Single-Trial EEG Classification

**DOI:** 10.1371/journal.pone.0135697

**Published:** 2015-08-21

**Authors:** Blair Kaneshiro, Marcos Perreau Guimaraes, Hyung-Suk Kim, Anthony M. Norcia, Patrick Suppes

**Affiliations:** 1 Center for the Study of Language and Information, Stanford University, Stanford, California, United States of America; 2 Department of Electrical Engineering, Stanford University, Stanford, California, United States of America; 3 Department of Psychology, Stanford University, Stanford, California, United States of America; University of Pécs Medical School, HUNGARY

## Abstract

The recognition of object categories is effortlessly accomplished in everyday life, yet its neural underpinnings remain not fully understood. In this electroencephalography (EEG) study, we used single-trial classification to perform a Representational Similarity Analysis (RSA) of categorical representation of objects in human visual cortex. Brain responses were recorded while participants viewed a set of 72 photographs of objects with a planned category structure. The Representational Dissimilarity Matrix (RDM) used for RSA was derived from confusions of a linear classifier operating on single EEG trials. In contrast to past studies, which used pairwise correlation or classification to derive the RDM, we used confusion matrices from multi-class classifications, which provided novel self-similarity measures that were used to derive the overall size of the representational space. We additionally performed classifications on subsets of the brain response in order to identify spatial and temporal EEG components that best discriminated object categories and exemplars. Results from category-level classifications revealed that brain responses to images of human faces formed the most distinct category, while responses to images from the two inanimate categories formed a single category cluster. Exemplar-level classifications produced a broadly similar category structure, as well as sub-clusters corresponding to natural language categories. Spatiotemporal components of the brain response that differentiated exemplars within a category were found to differ from those implicated in differentiating between categories. Our results show that a classification approach can be successfully applied to single-trial scalp-recorded EEG to recover fine-grained object category structure, as well as to identify interpretable spatiotemporal components underlying object processing. Finally, object category can be decoded from purely temporal information recorded at single electrodes.

## Introduction

Recognizing objects from different categories is of fundamental importance for survival. The human visual cortex has evolved efficient mechanisms for solving this problem, and a central goal of cognitive neuroscience has been to understand how this seemingly effortless feat is accomplished. A useful framing of the problem has been provided by DiCarlo and colleagues [1], who factor object recognition into component processes such as the segmentation of an object from its supporting background, and motor-action relations, such as object tracking, avoidance, and grasping—all of which are distinguished from “core object recognition,” which is defined as extremely rapid and accurate discrimination of object categories, independent of changes in position, size, pose, and background context.

An important component of understanding the core object recognition process has been the determination of its anatomical substrate. Object-selective regions in visual cortex have been identified in human neuroimaging studies on the basis of preferential responses to intact versus scrambled objects [2–4] (see [5, 6] for reviews). These regions comprise a large area of occipital cortex anterior and lateral to early visual cortex, subdivided into ventral-temporal and lateral-occipitotemporal regions [7]. Within these regions lie foci that are preferentially responsive to images of objects from different categories such as faces, houses, body parts, or scenes [8–10]. Such preferences are not absolute, however, as these regions contain information about multiple object categories [11–13].

A parallel line of research in humans has focused on the temporal evolution of category selectivity. Comparisons of the distributions of event-related potential (ERP) amplitudes, polarities, and scalp topographies have consistently found that scalp negative activity between approximately 120 and 200 ms depends upon object category, particularly for lateral scalp electrodes such as P7 and P8 [14–16]. Some studies have also reported differential scalp positive activity for objects from different categories at medial electrodes [17, 18], but this activity may have arisen from differences in low-level features such as color, contrast summary statistics, and Fourier amplitude [19–21]. Distributions of Fourier phase, rather than Fourier amplitude, appear to be the dominant stimulus features underlying the earliest category specificity in the ERP [21]. Differential evoked responses for images containing animate versus inanimate objects occur no later than 150 ms [22], and responses to faces show an earlier N1 response bilaterally with a stronger right-hemisphere response for parietal and parietal-occipital electrode locations [14, 17, 23], and equivalently near the vertex as the Vertex Positive Potential (VPP) [24–26].

Recent electroencephalography (EEG) and magnetoencephalography (MEG) studies have investigated category selectivity using multivariate pattern classification. This approach does not require preselection of spatial or temporal components of the brain response for analysis, but rather enables the full response to be analyzed at once [27–29], and can additionally provide a data-driven means of identifying spatial, temporal, and spectral components underlying category discrimination [30–32]. The first studies to use this approach classified single-trial EEG responses to photographs of faces and cars at different levels of phase coherence as a means of relating neural components to behavioral discrimination and task difficulty [30, 33]. Subsequent studies have demonstrated that up to six object categories—including photographs of faces, objects, and scenes [27, 28, 31, 32, 34–38], as well as line drawings and spoken and written names of items [29, 39]—can be decoded from multivariate EEG and MEG data as early as 80 ms after stimulus presentation. A recent study has also taken an encoding approach, predicting MEG activations from computational and semantic features of visual stimuli [40].

The fact that EEG/MEG-recorded responses contain information about object category has led to a fine-grained analysis of the representational structure of object categories [41]. The underlying notion of a representational structure, as operationalized by Representational Similarity Analysis (RSA) [42], is that responses across different modalities—for example, from a variety of behavioral and neural measures, computational models, and stimulus models—can be directly compared by considering not the responses themselves—which may be numerous, high dimensional, and of incompatible structure—but rather the pairwise distances between them, as can be summarized in a Representational Dissimilarity Matrix (RDM). The RSA approach was first used to compare the similarity structure of object recognition as represented by the spiking activity of single neurons in inferotemporal cortex of the macaque [43] with that observed in a large number of voxels in the presumptive human homologue of IT, as measured by functional magnetic resonance imaging (fMRI) [44]; the comparison between IT neuron and BOLD measurements was facilitated by the use of an overlapping stimulus set. Various measures have been used to derive the pairwise distances of the RDM, including pairwise correlations of responses and stimulus features [42, 44], pairwise classifier accuracies [36, 41], and pairwise differences in single data points of a response vector, such as time samples of the ERP [20].

A recent MEG study has used the same image set as Kriegeskorte and colleagues [44] to study both the structure and temporal evolution of the representational space. Using pairwise decoding of responses to individual image exemplars, Cichy and colleagues [41] found that exemplar-level information could be decoded with peaks around 100 ms, but that more abstract category information emerged later. The decoding matrices of this study indicate that decoding accuracy within an ordinate category—particularly the human face category—is poor at all latencies, but that within the superordinate category, faces and bodies cluster separately. The representational space derived from MEG shared common structure with V1 BOLD measurements at early latencies, with the structure shifting to be more like that of IT at longer latencies. A similar result was found by Carlson and colleagues [36] on the basis of 24 images over six categories.

For the present study we used a similar classification approach, but with EEG rather than MEG recordings. While the two measurements share common underlying sources, they have differential sensitivity to tissues of different orientations and depths [45]. The use of RSA and a stimulus set derived from one used previously [41, 44] allows a direct comparison of EEG and MEG representational structure, enabling us to determine points of similarity and difference between the two measures, and to further allow a comparison with representational structure derived from IT neurons and human fMRI measurements. Previous work on RSA has used dissimilarity metrics based upon pairwise correlations [42, 44] or pairwise classification accuracies [36, 41]. Here we use a slightly different approach to derive an RSA metric, constructing the RDM from the confusion matrix of a multi-class classification. We perform single-trial classification on both category and exemplar levels and examine the non-hierarchical and hierarchical structure of the resulting representational space. Temporal and spatial components of the brain response that successfully discriminate image categories and exemplars are identified. Finally, we focus our analysis on two commonly compared image categories—human faces and objects—and compare between-category and within-category object processing.

## Methods

### Ethics Statement

This research was approved by the Institutional Review Board of Stanford University as part of the study titled “Brain-wave recognition of language, music, and visual images using EEG recordings.” Written informed consent was obtained from each participant prior to his or her involvement in experimental sessions.

### Participants and Stimuli

Ten participants, aged 21 to 57 years (median age 30.5 years; 3 female; 1 left-handed), took part in the experiment. All participants reported normal color vision and either normal or corrected-to-normal vision.

**Fig 1:**
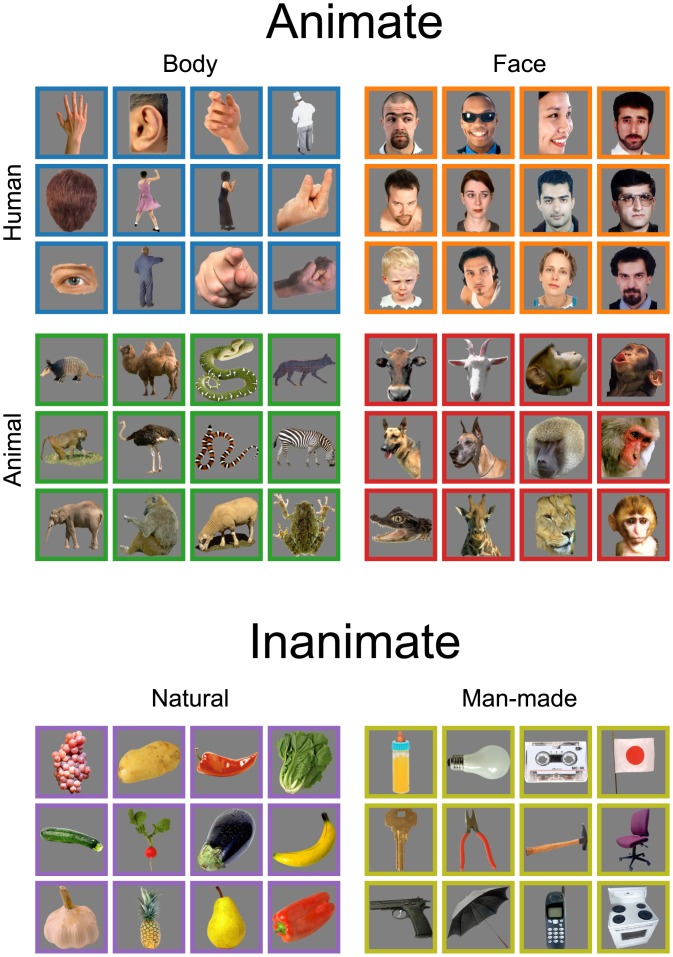
Stimulus set used in the experiment. The 72 images used in this study include twelve images from each of six categories: Human Body, Human Face, Animal Body, Animal Face, Fruit Vegetable, and Inanimate Object. The stimuli can be divided most broadly into Animate and Inanimate categories. Within the Animate category, images are either Human or Animal and Body or Face. Inanimate images are either Natural or Man-made. Colored borders are added for visualization purposes only, and were not shown during experimental sessions.

Seventy-two photographs of real objects were used as stimuli, taken from the 92-image set used in past RSA studies [41, 44]. We reduced the quantity of inanimate images in the set in order to ensure that each image category would contain the same number of exemplars—specifically, twelve images from each of the following six categories: Human Body (HB), Human Face (HF), Animal Body (AB), Animal Face (AF), Fruit Vegetable (FV), and Inanimate Object (IO). As illustrated in Fig 1, the stimuli can first be separated into Animate and Inanimate superordinates; Animate images can then be subdivided into four categories along dimensions of Human or Animal and Body or Face, while Inanimate images are either Natural (Fruit Vegetable) or Man-made (Inanimate Object). Color images were set against a mid-gray background with no border, spanning up to 7.0° × 6.5° of visual angle.

### Experimental Paradigm

Each participant sat in a chair in a darkened, acoustically shielded booth for the duration of the experimental sessions; no chin rest was used in the experiment. The chair was positioned in front of a desk such that the measured viewing distance to the monitor on the desk was 57 cm. Images were presented on a Dell 1905FP computer monitor with mean luminance of approximately 100 cd/m^2^. Each trial consisted of a single image, set against a mid-gray background matching the image background, shown onscreen for 500 ms, followed by 750 ms of a blank gray screen, for a total inter-trial interval of 1,250 ms. To minimize eye movements during and between trials, a white fixation cross spanning 0.76° × 0.76° of visual angle was superimposed on the center of each image, and in the same position on the blank screen shown between image presentations. Stimuli were presented using Neurobehavioral Systems Presentation software (http://www.neurobs.com) in blocks of 864 trials, in which each image was shown twelve times in random order. Short breaks were given after every 36 trials; longer breaks were given between blocks to check electrode impedances. Each participant completed two experimental sessions, each containing three blocks, spaced between six and eight days apart. In total, each participant completed 72 trials of each of the 72 images, for a total of 5,184 trials per participant.

### Data Acquisition and Preprocessing

Data were collected at the Center for the Study of Language and Information at Stanford University. Dense-array EEG was recorded using unshielded 128-channel EGI HCGSN 110 nets [46]. Data were acquired with vertex reference using the EGI Net Amps 300 amplifier and EGI Net Station 4.4 acquisition software, sampled at 1 kHz with a range of 24 bits. Electrode impedances at the start of each experimental block were within acceptable thresholds for this system, not exceeding 80 kΩ.

Data preprocessing was performed offline using Matlab software (http://www.mathworks.com). A high-pass fourth-order Butterworth filter removed frequency content below 1 Hz for removal of DC offset; this non-causal filter had a small effect on onset timing [47–49], but does not fundamentally impact the results presented here due to our use of 80-ms temporal smoothing windows, described below. A low-pass eighth-order Chebyshev Type I filter removed frequencies above 25 Hz before data were temporally downsampled by a factor of 16, for a final sampling rate of 62.5 Hz. Electrodes 1 through 124 were retained for further analysis, as were VEOG and HEOG channels for artifact removal. Ocular artifacts were removed from each participant’s EEG record using the extended Infomax ICA algorithm [50, 51] as implemented in the Matlab EEGLAB toolbox [52]. Cleaned data records were then converted to average reference. Data were epoched into trials using 32 time samples (496 ms) of post-stimulus response, time-locked to stimulus onset. As a final preprocessing step before classification, each space-time EEG data matrix *X*_0_, in which rows represent electrodes and columns represent time samples of voltage, was reshaped to a trial-space matrix *X*_1_, in which rows represent trials and columns contain concatenated time courses of all electrodes’ data pertaining to that trial. The trial-space EEG datasets used for classification are available for public download from the Stanford Digital Repository, http://purl.stanford.edu/bq914sc3730.

### Single-Trial Classification

In a classification task, a model is built from a set of labeled observations, and then used to predict labels of test observations. Each observation is represented by a set of features aggregated into a feature vector. For the present study, an observation is defined to be the EEG response pertaining to one experimental trial, and the feature vector describing each observation is the corresponding set of time-sampled voltages from electrodes and time points of interest. A label is a descriptor of the stimulus that was shown in a trial, and can be either an image name or image category.

When a feature vector incorporates data from multiple time points and electrodes, its dimensionality can become high relative to the number of trials collected. For example, the full set of 124 electrodes and 32 time samples per electrode in the present study produces a feature vector of length 124 × 32 = 3,968, compared to 5,184 observations per participant. Such a relatively high-dimensional feature vector may result in the classification model fitting to non-meaningful noise in the data. In addition, high correlations among data recorded from adjacent electrodes may cause redundancies in the data. To address these problems, we applied dimensionality reduction to the data matrix *X*_1_ using Principal Component Analysis (PCA), implemented using Singular Value Decomposition (SVD), whereby the trial-space data matrix *X*_1_ = *U*Σ*V* ^*T*^ is rotated into the component-space matrix *X*_1_ *V* = *U*Σ [53, 54]. The exact number of Principal Components used in a given classification was determined during cross validation, as described below. Henceforth we use the term “feature vector” to refer to the composition of the vector of EEG data to be used in a classification, prior to dimensionality reduction.

All classifications were performed within-participant using Linear Discriminant Analysis (LDA) [55]. Multi-class classifications were performed using a one-against-all strategy (see [56, 57] for details of classifier implementation). In order to have an unbiased estimation of the classification rates, we used a cross-validation procedure. Each classification used ten-fold cross validation [58, 59], whereby the data were randomized and partitioned into ten roughly equal-sized subsets, over which ten training-test iterations occurred. Each partition was used as the test set exactly once, with the remaining nine partitions used for training in that fold. In each fold, the nine training partitions underwent a nested ten-fold cross validation, and it was here that the number of Principal Components was optimized to a value *k* between 3 and min(200, *K*), where *K* denotes the total length of the feature vector. Once the optimal number of components was determined, classification was performed on the outer test partition using the *k*-dimensional reduction of the component-space data. Because it was computationally expensive to perform the SVD within each cross-validation loop, SVD was performed once on the data matrix *X*_1_ before it was partitioned into cross-validation folds. For comparison with LDA, we performed a subset of classifications using Support Vector Machine (SVM) with a radial Gaussian kernel [60], implemented using LIBSVM [61] and using the same component optimization procedures as the LDA classifications. LDA produced superior results, and was also approximately ten times faster to execute.

In addition to classifying the full response—incorporating data from all time samples and electrodes in the feature vector—we performed classifications on spatial and temporal subsets of the response in order to gain more insight into the dynamics of object category representation. For spatial resolution, independent classifications were performed using all time samples of the response from one electrode at a time. Doing so, each classification was purely temporal (no spatial variation), and resulting rates indicated which electrodes contained data that classified successfully (see [62] for discussion of temporal versus spatial classification). This analysis is analogous to the “searchlight” approach to RSA [63], and has recently been applied to MEG-sourced data [64]. To complement the spatial insight gained by independent temporal classifications, we performed separate classifications on overlapping consecutive temporal subsets of the response, six time samples (80 ms) long and advancing in three-sample (48-ms) increments. Each classification combined data from all 124 electrodes but comprised little temporal variation; therefore, these classifications were primarily spatial, providing insight into the temporal dynamics of category selectivity in the brain response over successive time windows. Finally, independent single-electrode classifications were performed for each temporal window described above in order to highlight changes in topography over time; here, each feature vector for classification contained only six time samples of EEG data prior to dimensionality reduction.

Each classification produces a confusion matrix, which summarizes classifier predictions for all test observations. For the present study, rows of the confusion matrix denote actual labels, while columns denote predicted labels. Element (*i*, *j*) of the confusion matrix thus expresses the number of observations having label *i* that the classifier labeled as *j*. Values on the diagonal (*i* = *j*), where actual and predicted labels are the same, denote correct classifications. Because all classifications in the present study involved a balanced number of observations per category, we may divide every element of the confusion matrix by the sum of its row so that every row sums to 1. Therefore, element *CM*_*ij*_ in normalized confusion matrix *CM* expresses proportions—rather than counts—of classifications, and *CM* can be interpreted as an estimated conditional probability matrix [65]. We henceforth use the term “confusion matrix” to refer to this normalized confusion matrix *CM*. Classifier accuracy, defined as the percentage of classification attempts that correctly predicted the label of the observation, is thus computed as the mean of the diagonal of the (normalized) confusion matrix *CM*. We report mean within-participant accuracies across the ten participants.

### Multidimensional Scaling and Clustering

Misclassifications among stimuli can be interpreted as measures of proximity or similarity [66, 67]. Along these lines, the misclassifications contained in off-diagonal elements of a confusion matrix can be converted from similarity measures to distance measures, which can then be used for RSA [42]. As our analysis used multi-class classifiers, by which the classifier could choose among all *N* available categories for assigning predicted labels to observations, diagonal elements of the confusion matrix were not undefined, as is the case for pairwise classifications; nor were they zero, as is the case for 1 − *ρ* distance measures used in correlation-based analyses. Therefore, our first step in converting the confusion matrix to an RDM was to normalize the confusion matrix. This was accomplished by dividing each matrix entry by the diagonal of its respective row, *CM*_*ij*_ = *CM*_*ij*_/*CM*_*ii*_, which served to achieve a unity measure of self-similarity for each label [68]. Following this, we computed mean similarity measures for each pair of labels by computing the geometric mean of the matrix and its transpose, $S=\sqrt{(}CM\times CM^{T})$[68]. Finally, the distance vector *D* was calculated from the lower diagonal of the matrix, *D* = ℒ(1 − *S*).

To assess the representational structure of the stimuli, we applied two exploratory data visualization methods to the set of distance vectors: Classical multidimensional scaling (MDS) and hierarchical clustering. We applied classical MDS to *D*, converting the pairwise distances to coordinates whose orthogonal dimensions are sorted in order of descending variance [69–71]. The resulting coordinates were projected down to two principal dimensions at a time for visualization purposes. Hierarchical clustering was performed on *D* using unweighted pair grouping method with averaging (UPGMA) for linkage [72], and results were visualized as dendrograms.

### Statistical Analyses

Along with classifier accuracies, we report statistical significance, effect size, and sample standard deviation. As all classifications were balanced, with equal numbers of observations in each class, statistical significance was calculated under the null hypothesis of the Binomial distribution, using as parameters the floored mean correct classifications in one test fold; number of classifications attempted in one test fold; and chance-level accuracy of 1/*n* *Categories*. No multiple comparison correction was made for temporally and spatially resolved classifications. As a measure of effect size, we report *d* = (*C*_*obs*_ − *C*_0_)/*σ*, the number of standard deviations between the observed and chance-level number of correct classifications [73], calculated again under the null hypothesis of the Binomial distribution. Finally, as a measure of variability of classifier accuracies across the participant population, we report the sample standard deviation across participants, computed using the unbiased estimator of population variance.

For analysis of MDS plots derived from the 72-class confusion matrix, we performed two-tailed Wilcoxon rank-sum tests [74, 75] on the MDS coordinates of image exemplars (grouped by planned category) along a given dimension, in order to determine whether that dimension successfully separated the exemplars into the planned categories.

## Results

### Category-Level Classifications

**Fig 2:**
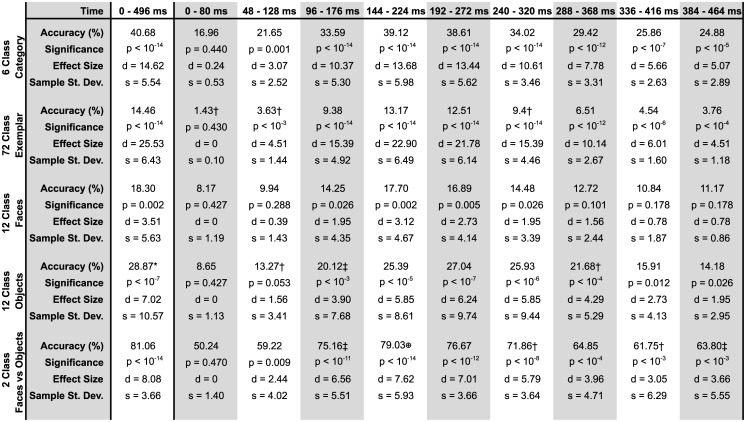
Summary table of classification results. Classifier accuracies, along with *p*-value (p), effect size (d), and sample standard deviation (s) across the ten participants, for classifications incorporating data from all electrodes into the feature vector. Classifications using all time points together are shown in the “0–496 ms” column. Temporally resolved classifications are shown in subsequent columns. Chance level for six-class (category-level) classifications was 1/6 = 16.67%; for 72-class (exemplar-level) was 1/72 = 1.39%; for twelve-class (within-category) was 1/12 = 8.33%; and for two-class (between-category) was 1/2 = 50.00%. Statistical significance and effect size were calculated under the null distribution of the Binomial distribution based upon the number of observations in one test fold. Some classifications could not be performed for certain participants’ data due to the SVD not converging in the computation of Principal Components. Results are from all ten participants unless otherwise indicated: † indicates nine participants; ‡ indicates eight participants; ⊕ indicates seven participants. A ⋆ indicates missing data from one participant in our results file.

**Fig 3:**
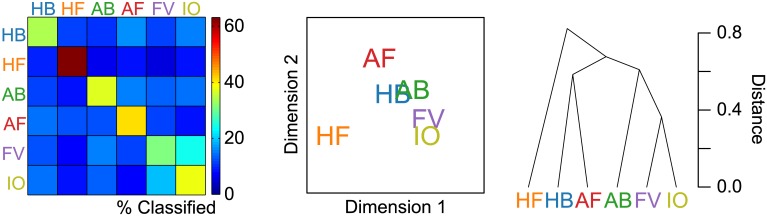
Category-level classification results. All electrodes and time samples of the brain response were used together in the six-class category-level classification. An equal number of observations from each category were used. Left: Confusion matrix showing proportions of classifier output. Rows represent actual labels and columns represent predicted labels. Values along the diagonal indicate proportions of correct classifications. Mean accuracy for this classification was 40.68%, compared to chance-level accuracy of 16.67% (Fig 2). Middle: Multidimensional scaling (MDS) plot derived from the confusion matrix, visualizing the non-hierarchical structure of the representational space. MDS dimensions are sorted in descending order of variance explained. Right: Dendrogram visualizing the hierarchical structure of the representation. The Human Face category is most separate from the other categories, while the two Inanimate categories form the tightest category cluster.

Initial classifications were performed at the category level, meaning that each EEG trial was labeled with the category—rather than the exemplar name—of its corresponding stimulus. Chance-level accuracy for this six-class classification was 1/6 = 16.67%. When the feature vector included the full response—all electrodes and time points together—mean classifier accuracy was 40.68% (*p* < 10^−14^, *d* = 14.62, *s* = 5.54), as summarized in Fig 2. The confusion matrix, MDS plot, and dendrogram corresponding to this classification are shown in Fig 3. As the highest value in each row of the confusion matrix lies along the matrix diagonal, labels predicted by the classifier were most often the correct category labels, for all six categories. The Human Face category classified most succesfully, with 63.14% of trials labeled correctly. As can be seen in the lower right portion of the confusion matrix, the two Inanimate categories show notable confusion. The MDS plot shows the separability of the object categories along the principal (x-axis) and secondary (y-axis) dimensions. Along Dimension 1, the Human Face category is well separated, while Animal Face and Human Body share similar coordinates, as do Animal Body, Fruit Vegetable, and Inanimate Object. Dimension 2 to some extent separates the members within each of these latter two category clusters. Finally, the hierarchical structure of the categories is made explicit in the dendrogram. The top cut in the dendrogram separates Human Face from the other categories, followed by a Human Body/Animal Face cluster. As could be inferred from the confusion matrix, the two Inanimate categories form the tightest category cluster.

**Fig 4:**
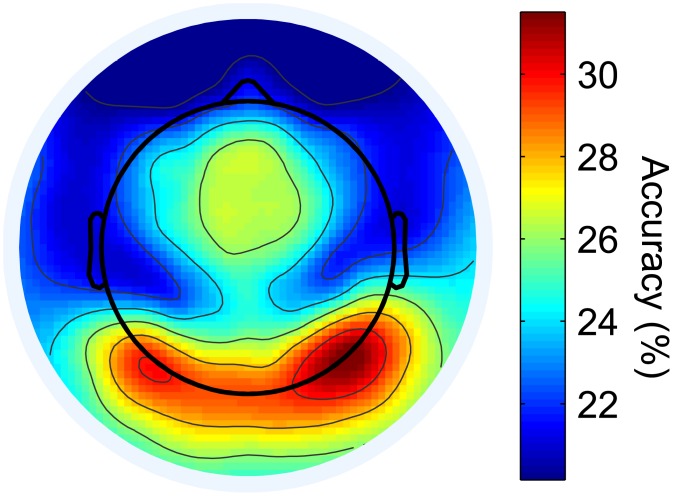
Topographic map of category-level classifier accuracies for individual electrodes. Independent category-level classifications were performed on the data from each of the 124 electrodes, and resulting classifier accuracies were plotted over a scalp map. Accuracies of 20.66% or higher are statistically significant at the *α* = 0.01 level.

To investigate the topography of object category separability, we next performed spatially resolved six-class classifications. Resulting accuracies are plotted over a scalp map in Fig 4. Mean accuracies from individual electrodes range from 20.15% (*p* = 0.018, *d* = 2.12, *s* = 1.41) at frontal locations to 31.51% (*p* < 10^−14^, *d* = 9.08, *s* = 2.72) at electrode P8, part of a cluster of high-performing electrodes located above right lateral occipital cortex. Other regions of above-chance classification include medial and left lateral occipital cortex, as well as near the vertex. Given that category-level classifications were found to be driven primarily by the Human Face category, it makes sense that the topography of individual-electrode classification rates is consistent with previous EEG studies indicating that the face-related N1 component is larger over this region [17, 26], and also with fMRI BOLD results [44].

**Fig 5:**
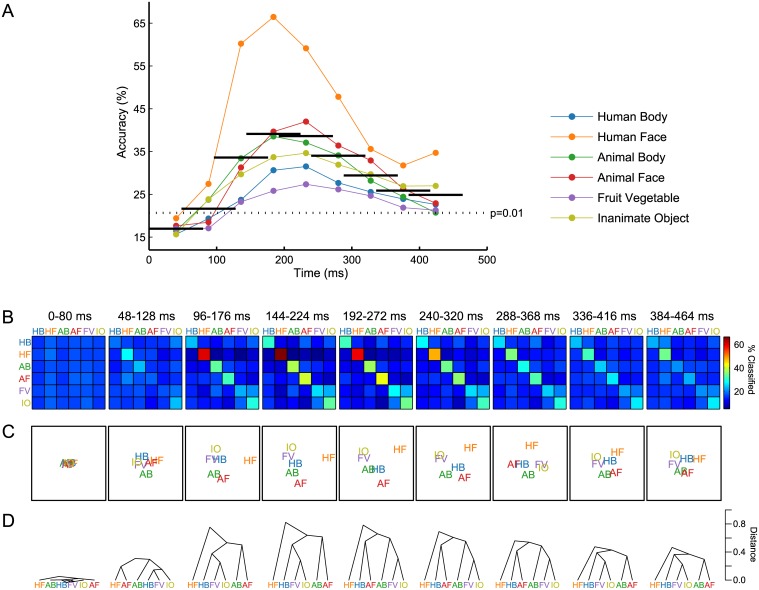
Temporally resolved category-level classification results. Separate classifications were performed on temporal subsets of the brain response. Each temporal window was 80 ms long and advanced in 48-ms increments. Classifications used data from all electrodes together. Chance level was 1/6 = 16.67%. (A) Classifier accuracies as a function of time for the nine temporal windows. The width of each black bar specifies the time interval used in classification, and the height indicates the overall accuracy of that classification. The line plots through the center of each temporal window display proportions of correct classifications for each of the six categories. The dotted horizontal line indicates the statistical significance threshold of the overall classifier accuracy at *α* = 0.01. Peak accuracy in the fourth temporal window (144–224 ms) is 39.12%, compared to 40.68% accuracy when all time points were used together in classification (Fig 2). (B) The nine confusion matrices corresponding to the classifications performed in (A). Composition of the matrices is as described in Fig 3. The diagonal values of each confusion matrix make up the line plots in (A). (C) MDS plots derived from the confusion matrices in (B). As in Fig 3, Dimension 1 of the MDS is plotted along the x-axis, and Dimension 2 is plotted along the y-axis. (D) Dendrograms derived from the confusion matrices in (B).

Temporally resolved classifications combining data from all electrodes together produced classifier accuracies plotted as a function of time in Fig 5A. The widths of the black horizontal bars denote the time intervals used in each classification, and heights are the corresponding classifier accuracies. Values along the diagonal of each confusion matrix are plotted at the center of each time interval. The earliest temporal window of the EEG response (0–80 ms) does not contain category-discriminating information, and classifies around chance level (16.96%; *p* = 0.440, *d* = 0.24, *s* = 0.53). However, classifier accuracies become statistically significant as early as the second temporal window spanning 48–128 ms (21.65%; *p* = 0.001, *d* = 3.07, *s* = 2.52), reaching peak accuracy of 39.12% in the 144–224 ms window (*p* < 10^−14^, *d* = 13.68, *s* = 5.98), which is the time interval containing the N1 component. This peak accuracy approaches the accuracy obtained using the full time course of the response (Fig 3). Aggregate classifier accuracies decrease monotonically over subsequent temporal intervals, but remain statistically significant. As can be appreciated from the plot, classification success is driven primarily by the Human Face category, which also shows an increase in classification success in the late stage of the response. Corresponding confusion matrices, MDS plots, and dendrograms, shown in Fig 5B-5D, show a lack of category structure in the earliest stage of the response, followed by a face/non-face distinction in the second temporal window, which may reflect low-level feature differences driving the P1 component [19]. From the third temporal window (96–176 ms) on, Human Face becomes the most distinct category, while the two Inanimate categories form the tightest category cluster. The hierarchical arrangement of the remaining Animate categories varies throughout the response.

**Fig 6:**
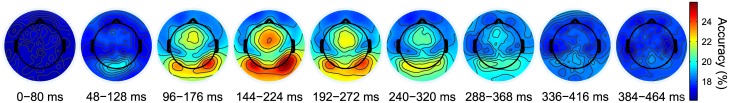
Temporally resolved rate maps for single-electrode category-level classifications. For each overlapping temporal window described in Fig 5, independent category-level classifications were performed on single-electrode data (as described in Fig 4) in order to reveal the topography of the representation over time. Resulting classifier accuracies are plotted over separate scalp maps for each temporal window. 20.66% is the accuracy threshold for statistical significance at *α* = 0.01.

The final category-level classifications combine spatial and temporal resolution. Classifier accuracies from individual electrodes in overlapping temporal windows are plotted in the scalp maps shown in Fig 6. As with previous classifications, no significant accuracies are revealed in the first temporal interval. In the 48–128 ms temporal interval, the region underlying medial occipital electrodes classifies above chance, though not significantly. Based upon the clustering of Human Face and Animal Face categories in the MDS plot of the all-electrode classification during this temporal window (Fig 5), we reason that these electrodes classify based upon responses to low-level features shared by faces [19]. From 96–272 ms, the topography of classifier accuracies is similar to classification using the full response (Fig 4), with overall maximal classification in the right occipital region from 144–224 ms—the same temporal window that produced highest classifier accuracies when using all electrodes together in a given temporal interval (Fig 5). In sum, the spatiotemporal results concur with spatial and temporal findings separately; however, we note that scalp topographies for final temporal intervals (336–464 ms) do not reveal specific regions of high classifier accuracy, though classifications using all electrodes did produce above-chance results at these times (Fig 5).

### Exemplar-Level Classifications

The category-level classifications performed thus far faciliated examination of spatial and temporal components of the brain response that differentiate visual objects according to *a priori* natural language categories used in previous studies. The suitability of such category labels, however, was not always substantiated; for example, our category-level results suggest that the two Inanimate categories may in fact belong to a single superordinate. At the same time, pre-assigned category labels preclude the emergence of spontaneous category structures that may exist across or within those six categories. Therefore, we performed exemplar-level classifications, in which the classifier attempted to identify the specific image the participant was viewing, not just the category to which it belonged. Chance-level accuracy for exemplar-level classifications was 1/72 = 1.39%.

**Fig 7:**
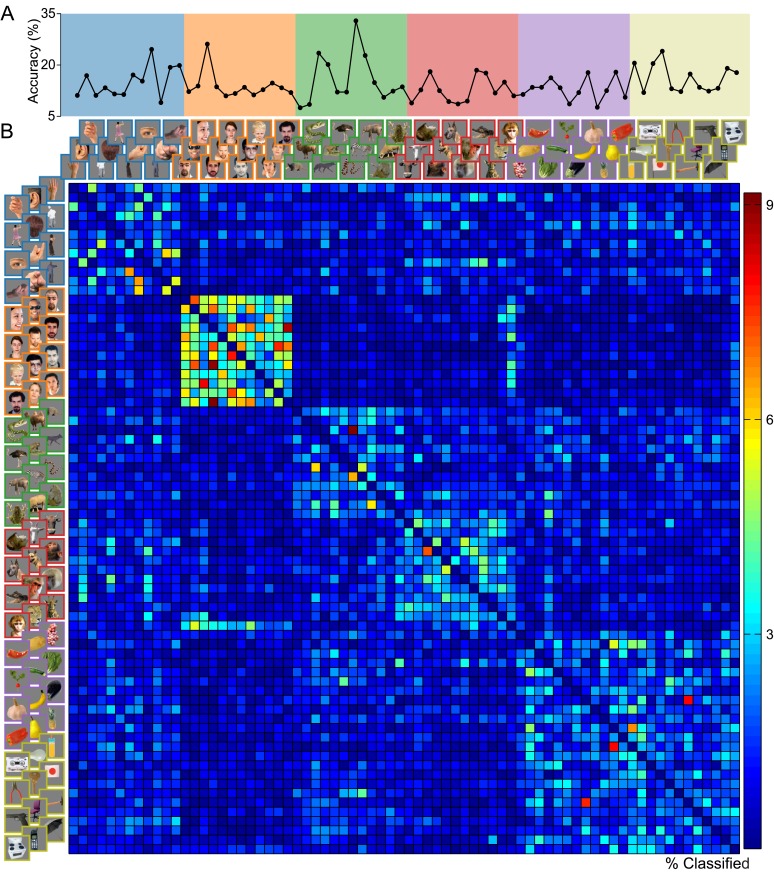
Exemplar-level classification results. The classifier attempted to predict image exemplar labels from brain responses in a 72-class classification. Mean accuracy for the classification was 14.46%. (A) Line plot of the proportion of correct classifications for each of the 72 image exemplars. (B) Confusion matrix from the classification. The matrix diagonal, visualized in (A), has been set to zero for better display of off-diagonal elements.

Exemplar-level classification using data from all electrodes and time samples together produced a mean accuracy of 14.46% (*p* < 10^−14^, *d* = 25.53, *s* = 6.43) as summarized in Fig 2. The plot of individual exemplar accuracies (Fig 7A) indicates that decodability of the EEG responses varied by image exemplar. The off-diagonal elements of the confusion matrix, representing misclassifications, are shown in Fig 7B. Here we see some evidence in support of the *a priori* categories used in the six-class classifications. The most visible category cluster involves the set of Human Face images, meaning that misclassified Human Face trials were most often assigned labels of other Human Face exemplars. A primate face image from the Animal Face category also shows high confusion with the Human Face images. While Human Face images, when grouped together, dominated the category-level classifications, exemplar-level confusions now emerge in the 72-class case. An exception is the profile face image, which is among the top-classifying exemplars. The other Animate images appear to exhibit within-category confusion, though to a lesser degree; and as was the case previously, the two Inanimate categories appear to be subsumed under a single superordinate.

**Fig 8:**
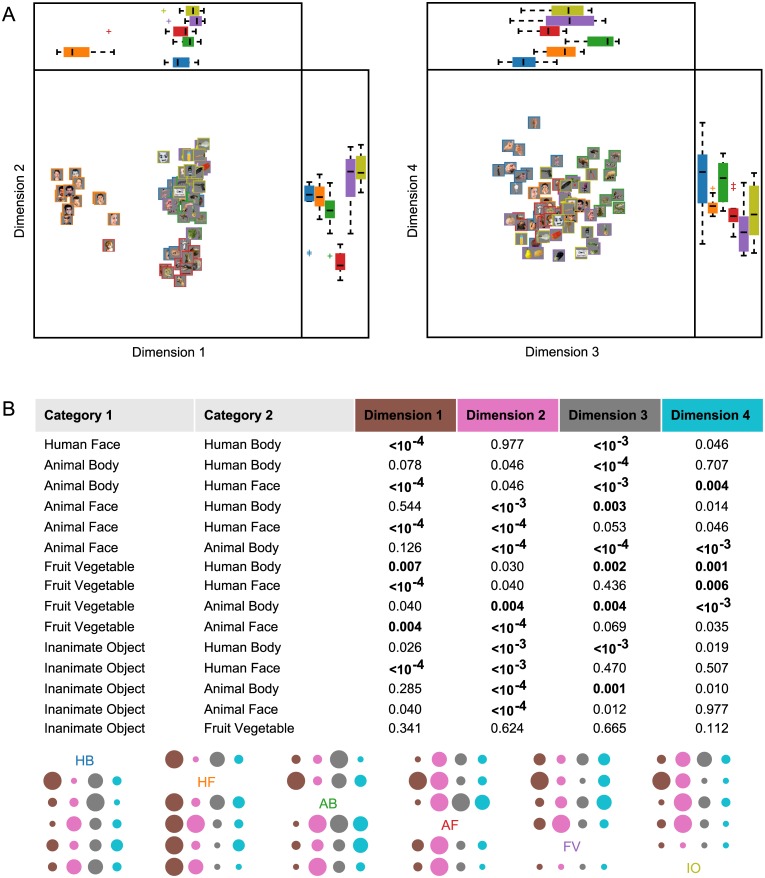
Multidimensional scaling plots for exemplar-level classification. MDS coordinates were derived from the 72-class confusion matrix (Fig 7). (A) The first four MDS dimensions are scatterplotted in pairs of dimensions. Boxplots show the distribution of image exemplar coordinates along each dimension, grouped by the category labels used previously (as in Fig 3). (B) Statistical significance of category separability along each of the four principal MDS dimensions plotted in (A). Nonparametric tests were performed on exemplar coordinates for MDS Dimensions 1–4 to assess category separability; all category pairs except for the two Inanimate categories are separable at the *α* = 0.01 level along at least one of the four principal MDS dimensions.

Dimensions 1–4 of the MDS plots corresponding to the 72-class confusion matrix are shown in Fig 8A. As was the case in the six-class MDS plot, Dimension 1 serves primarily to separate the effective Human Face images (including the primate face) from all other images. Dimension 2 separates Animal Face images, while Dimension 3 separates Animal Body and Human Body images. The MDS for Dimensions 3 and 4 also reveals evidence of sub-categories such as hands, bipedal/quadrupedal animals, and snakes. The boxplots accompanying the MDS plots display the coordinates of the image exemplars along each dimension, grouped by planned category label. We performed two-tailed Wilcoxon rank-sum tests on the coordinates in order to determine whether the images were separable by *a priori* category along the four principal MDS dimensions. Results, shown in Fig 8B, indicate that all category pairs could be statistically significantly separated along at least one of the MDS dimensions, with the exception of the two Inanimate categories, which failed to be separated with even marginal statistical significance (*α* ≤ 0.1) along any of the dimensions. This lends further support to the notion of a single Inanimate category.

**Fig 9:**
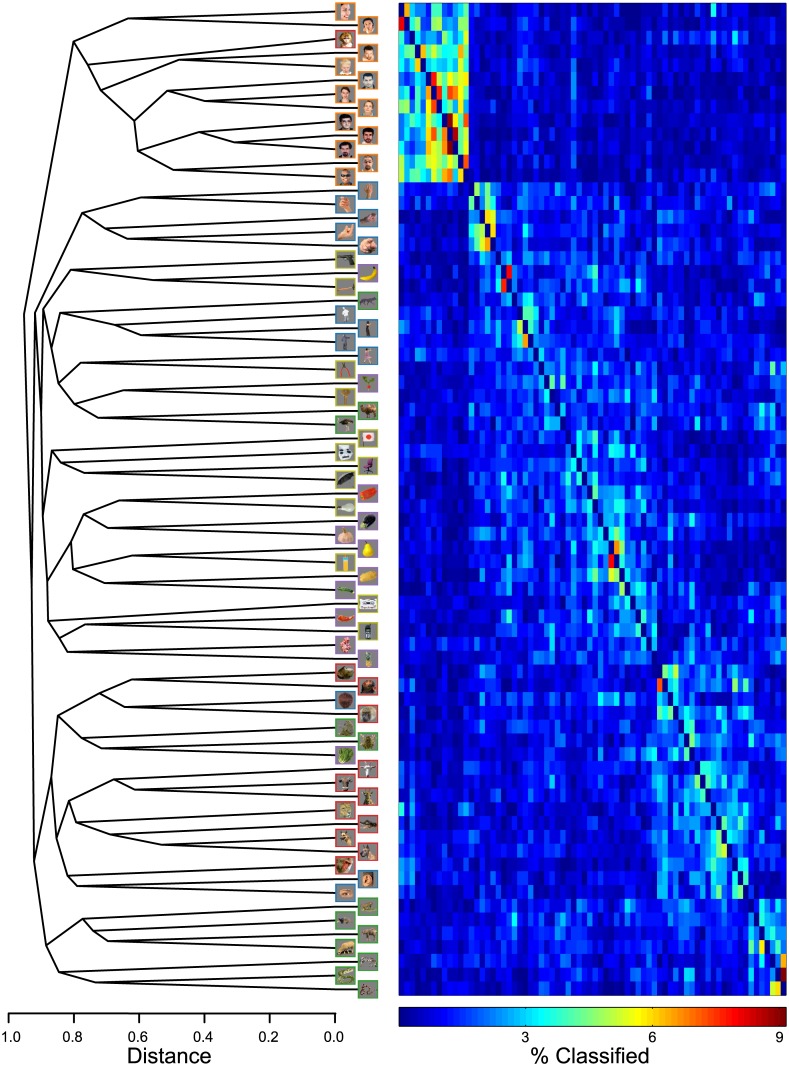
Dendrogram and reordered confusion matrix for exemplar-level classification. The dendrogram was derived from the 72-class confusion matrix shown in Fig 7. The principal split in the dendrogram separates Human Face images from the other images. To the right of the dendrogram is the 72-class confusion matrix, whose rows and columns have been reordered to match the ordering of elements in the dendrogram.

The hierarchical structure of the 72-class results are shown in the dendrogram in Fig 9. We observe distinct categories for Human Face images, while Animal Face images appear to have organized by orientation and combined with selected images from other categories. As the MDS plot suggested, we observe distinct sub-clusters for hands, snakes, and even standing Human Body images. To the right of the dendrogram is the confusion matrix, whose rows and columns have been reordered to reflect the structure of the dendrogram. Reorganizing the confusion matrix in this manner reveals the recovered category structure, which was somewhat obscured by the original row and column ordering.

**Fig 10:**
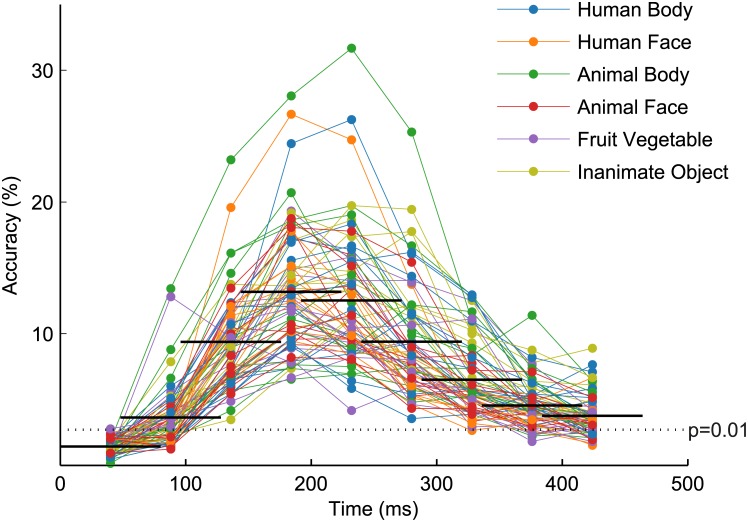
Temporally resolved exemplar-level classification results. Exemplar-level classification rates are plotted as a function of time. The temporal windows used for classification, and the composition of the plot, are as described in Fig 5. As in the category-level case (Fig 5), peak accuracy occurs in the fourth temporal window (144–224 ms). Here, exemplar-level peak accuracy is 13.17%, compared to 14.46% when all time samples were used at once (Fig 2).

Temporally resolved exemplar-level classifications were performed using data from all electrodes together. Classification rates as a function of time are plotted in the horizontal bars of Fig 10, with accuracies of individual exemplars plotted at the center of each temporal window. Mean classifier accuracies reach statistical significance as early as the second temporal window and remain significant through the final temporal window. As in the six-class case, peak accuracy is achieved in the 144–224 ms temporal window, at 13.17% (*p* < 10^−14^, *d* = 22.90, *s* = 6.49). However, where in the category-level classifications the Human Face category boosted the temporally resolved accuracies relative to the other categories, here we see exemplars from a variety of categories producing the highest classifier accuracies over time. This follows from the exemplar-level results presented in Fig 7, which revealed substantial confusion among Human Face exemplars.

### Within-Category Classifications

Human Face images have produced high category-level accuracy in the six-class case, and high within-category confusion in the 72-class case; both of these results point to a strong categorical representation for the Human Face exemplars used in this study. In contrast, the Inanimate Object images showed less category-level coherence and may in fact belong to a more general Inanimate category. Due to the contrasting representations exhibited by these two categories, we performed exemplar-level classifications *within* each category in order to investigate possible differences between category differentiation (is it a face or an object) and exemplar identification within a category (which face or object is it). Thus, we performed within-category Human Face classifications, and also within-category Inanimate Object classifications. As a basis for comparison, we performed two-class Human Face versus Inanimate Object classifications.

**Fig 11:**
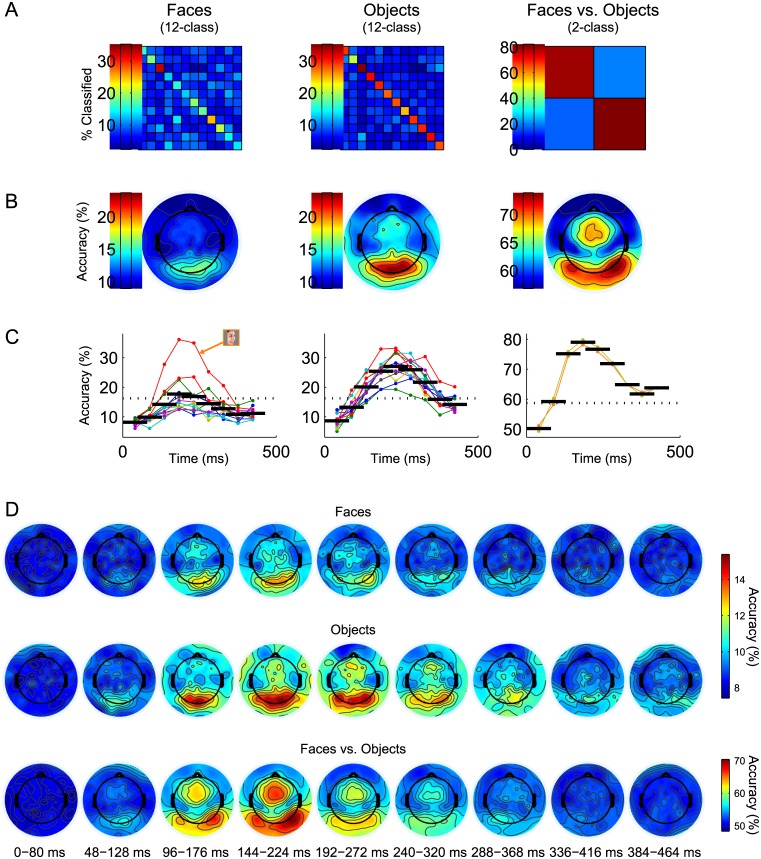
Within-category classification results. Separate within-category classifications were performed on Human Face and Inanimate Object responses. A two-class face-versus-object classification was also performed for reference. (A) Confusion matrices from classifications using data from all electrodes and time points together for Human Face (Left), Inanimate Object (Center), and face versus object (Right). Matrix layout is as described in Fig 3. Classifier accuracy for Human Face was 18.30%; for Inanimate Object was 28.87%; and for face versus object was 81.06% (summarized in Fig 2). (B) Maps of single-electrode classifier accuracies for Human Face (Left), Inanimate Object (Center), and face versus object (Right). Significance thresholds (*α* = 0.01) are 16.28% for twelve-class and 58.72% for two-class. (C) Classification rates as a function of time, with exemplar or category accuracies overlaid (as described in Fig 5) for Human Face (Left), Inanimate Object (Center), and face versus object (Right). Dotted horizontal lines designate *α* = 0.01 significance thresholds. The best-classifying Human Face image (Left) is highlighted. (D) Classifier accuracy maps for temporally resolved single-electrode Human Face (Top), Inanimate Object (Middle), and face-versus-object (Bottom) classifications, using spatiotemporal subsets described in Fig 6.

Fig 11A shows the confusion matrices resulting from classifications using data from all electrodes and time points combined. The mean classification rate for Human Face images was 18.30% (*p* = 0.002, *d* = 3.51, *s* = 5.63), and for Inanimate Object images was 28.87% (*p* < 10^−7^, *d* = 7.02, *s* = 10.57); chance level for twelve-class classifications was 1/12 = 8.33%. The within-category confusion of the Human Face images, observed previously in the 72-class classification, is seen again here, while Inanimate Object exemplars classify more successfully. In comparison, the between-category classification produces an accuracy of 81.06% (*p* < 10^−14^, *d* = 8.08, *s* = 3.66) compared to chance level of 1/2 = 50.00%, and has higher statistical significance, as well as lower inter-subject variance, than either of the twelve-class classifications.

Plots of single-electrode classifier accuracies, shown in Fig 11B, implicate medial occipital electrodes in the differentiation of exemplars within a category. This is in contrast to the bilateral occipital and vertex topography that best differentiates between the two categories. These results suggest that responses to low-level feature differences are used to differentiate the within-category exemplars used here, while face-specific components (such as the N1) may drive between-category classification.

Temporally resolved classification rates, using data from all electrodes together, are shown in Fig 11C. Within-category Human Face classifications achieve statistical significance (accuracies > 16.28%) from only 144–272 ms of the response, and not by a wide margin. Furthermore, the plots of exemplar accuracies indicate that the overall success of this classification is due to the one Human Face image in profile orientation. The peak accuracy for this classification occurs from 144–224 ms, with accuracy of 17.70% (*p* = 0.002, *d* = 3.12, *s* = 4.67). Classification of Inanimate Object exemplars is more successful, with statistically significant accuracies from 96–368 ms. Peak accuracy of 27.04% (*p* < 10^−7^, *d* = 6.24, *s* = 9.74) for this category occurs slightly later, from 192–272 ms. Between-category classifications become statistically significant in the 48–128 ms temporal window and persist through the remainder of the response. The temporal window producing peak accuracy of 79.03% (*p* < 10^−14^, *d* = 7.62, *s* = 5.93) is the same as the category-level peak window, 144–224 ms.

Fig 11D contains the temporally resolved single-electrode classification rates. The medial electrodes implicated in individual-electrode classifications across all time for within-category analysis (Fig 11B) are among the top-classifying cluster of electrodes in the best-classifying temporal windows (144–224 ms for Human Faces; 192–272 ms for Inanimate Objects). Interestingly, these peak intervals occur later than the P1, which was presumably the component represented by these electrodes in the category-level classifications (Fig 6). Thus, it may be the case that processing of low-level features persists in the brain response, and at these later time points becomes useful to the classifier for discriminating the image exemplars within each category. In contrast, the topographies of between-category classification rates quite closely resemble the voltage topographies in response to viewing an image of a human face [17].

## Discussion

A growing set of studies utilizing a shared image set across a variety of imaging modalities has shed light upon the processing and representation of visual object categories in the brain [36, 41, 43, 44, 76]. We have extended this research into the realm of EEG, and have shown that the first 500 ms of single-trial EEG responses to viewing a set of 72 images contains information allowing for successful category and exemplar decoding. A planned, hierarchical stimulus structure was used to investigate face and body categories of humans and animals, as well as natural and man-made object categories. Confusion matrices from the classifications were used to create a similarity space of the stimulus categories and exemplars. In the representational space derived from category- and exemplar-level classifications, the Human Face category proved to be the most coherent and distinct category. In contrast, the two Inanimate categories were least distinguishable from one another, forming essentially a single superordinate. We employed a searchlight approach, performing separate classifications on temporal and spatial subsets of the EEG response, to uncover the dynamics of object category processing. Classification accuracies were highest when the classifier had access to data from all electrodes (spatial pattern) and time samples (temporal pattern) of the response. However, classifications could be performed successfully using as little as 80 ms of response taken from a single electrode, represented by six time samples of EEG data. Results from these searchlight analyses suggest that both temporal and spatial dynamics of object category processing can be derived from scalp-recorded EEG responses.

### Temporal Evolution of Category Selectivity

Changes in object category representation over the time course of the brain response were investigated by performing separate classifications on overlapping temporal windows of the EEG response. We found that both object categories and image exemplars could be classified significantly above chance as early as 48–128 ms after stimulus presentation. Our results are roughly consistent with past EEG/MEG results reporting discriminability by object category as early as 80–100 ms [36] and around the P1 latency (for a different stimulus set [28]); and also with Cichy and colleagues [41], who report successful discrimination as early as 51–61 ms. Category- and exemplar-level classifications reached peak accuracy from 144–224 ms and remained statistically significant through the final 384–464 ms interval. Peak decoding times, too, concur with findings from past studies [28, 36, 41]. We note that the time course of the overall classifier accuracies does not always reflect the temporal evolution of decoding individual categories or exemplars, and in fact, there is notable variation in peak classification times of constituent elements (Figs 5 and 10).

### Structure of the Representational Space

Our current results point to a representational space dominated by a Human Face/non-Human Face distinction. The category-level classification using the full EEG response revealed the Human Face category to be most distinct from the other categories (Fig 3). This coherence and distinctness was evident even when the constraints of category labels were removed, as revealed by the separation of Human Face exemplars in the principal MDS dimension (Fig 8A), and the spontaneous top category formation of Human Faces in the dendrogram (Fig 9) derived from the exemplar-classification confusion matrix. For the dendrogram in particular, it is interesting to note that in the absence of category labels, many images tended to form sub-category clusters based upon attributes like orientation, pose, body part, and species, at lower levels of the dendrogram. This was not true for the Human Face images, which collectively formed the top cut in the dendrogram. The Human Face blocking observed in the 72-class confusion matrix (Fig 7) is comparable to the MEG decoding matrix shown by Cichy and colleagues [41], but differs from the human fMRI and monkey IT-derived RDMs [44], which displayed strong off-diagonal similarities for Human Face and Animal Face images and produced dendrograms with top category splits at the Animate/Inanimate level, while Human Face and Animal Face exemplars clustered at no higher than the tertiary split in the tree.

Category-level classifications additionally revealed the evolution of the representational space over time. As shown in Fig 5C and 5D, a tight, indistinguishable category structure exists at the very early stage of the brain response. Following this, a face-specific category cluster emerges in the dendrogram in the temporal window containing the P1, followed by the Human Face distinction we observed when performing classification on all time points together. While the grouping of the other categories changes somewhat over time, we do not observe a principal Animate/Inanimate split emerging later in the response, as Carlson and colleagues did [36], although that is not to say that the time course of such two-class classifications would not have followed a similar trajectory, had we performed them.

The present analysis used a multi-class classifier, meaning that within a given classification, each test observation was assigned exactly once to one of *N* ≥ 2 possible labels. As a result, confusion matrices contained counts (or proportions) of correct classifications along the diagonal, which varied in relation to a class’s overall discriminability. This is a novel source of information compared to previous RSA studies, which employed pairwise correlations (for which the diagonal was always 1 [44]) or pairwise classifications (for which the diagonal was undefined [36, 41]). By making use of this added source of data in deriving an RDM [68], our MDS and dendrogram plots not only reflect distances between elements in a space, but also show the overall size of the space, which, as we have found, varies depending upon the overall accuracy of the classification (as in Fig 5C and 5D).

### Spatial Topography of Object Classification

Accuracies from independent classifications of data from individual electrodes were plotted on the scalp in order to give insight into the topography of object category representation. When all time points of the response were used for classification, occipital and vertex electrodes contained the most discriminable information (Fig 4). When single-electrode classifications were performed across smaller temporal windows of the response, medial occipital electrodes were useful for early category discrimination (starting around 80 ms), followed by lateral occipital and vertex electrodes, which reached maximal accuracies in the 144–224 ms temporal window encompassing the N170 (Fig 6), similar to the results of Simanova and colleagues [29].

There is considerable evidence for spatial clustering of object preferences in occipitotemporal cortex [77–79] (see [80] for reviews). The animate versus inanimate dimension is reflected in regional differences in BOLD response, with images of animate categories such as faces being represented in lateral fusiform cortex [10] and inanimate categories such as scenes [9] and tools [81] represented more medially. Within the animate category, there is a further gradient of representation with primate images being represented in lateral fusiform and STS, whereas activity associated with insects was relatively greater in middle fusiform and lingual gyri, thus resembling the animate versus inanimate continuum [82]. Previous fMRI studies have found an alternating layout of face- and body-preferring regions in ventral-temporal cortex [79].

We find these categories to be well separated in terms of their evoked responses, at the category level for Human Face images, and in sub-category structures for the other Animate categories (Fig 9). Spatial gradients and clusters of category-preferring neurons may contribute, in part, to the topographic differences in the evoked response across object categories. However, with the category images used in the present study, there also exists a temporal dimension along which object categories can be separated. Whether temporal properties form a distinct signature of different object categories, or simply reflect low-level feature differences, cannot be determined from the present data, but will be an interesting topic for future research.

A number of previous studies have published scalp maps of the classifier weights/data features that are discriminative of object category [28, 29, 36]. These maps often have very fine features that are not plausibly relatable to intra-cranial sources being viewed at a distance through a low-conductivity skull. Rather, these types of scalp topographies are determined by an unknown admixture of signal and noise, though they can be transformed into physiologically meaningful maps through the use of a forward model [83]. Alternatively, the rates—rather than the weights—can be plotted, as we have done here. The topographic maps of classification rates utilized in this study are smooth and have a spatial distribution similar to maps that have been generated at the N1/N170 latency in transient ERP studies of face and object processing [14, 21, 28, 36, 84]. These scalp maps are consistent with activity in lateral occipital areas known to be associated with object-level processing [84].

### Between- Versus Within-Category Classifications

We further investigated object category representation by classifying image exemplars from what we considered to be the most coherent category (Human Faces) as well as a less coherent category (Inanimate Objects). Inanimate Object exemplars classified better than Human Face exemplars, a result that is consistent with the within-category decoding of Carlson and colleagues [36]. Rate maps from within-category classifications for both categories implicated electrodes closer to medial occipital cortex (Fig 11), suggesting that processing of low-level features was used to differentiate the image exemplars. These medial sites continued to drive classification at time points later than those used for between-category classifications, suggesting that classification of within-category exemplars relied here upon processing of subtle, higher spatial frequency feature differences in early areas. This is in contrast to previous studies on face identification, which have highlighted right occipital cortex and anterior inferotemporal cortex as playing a role in identification, as opposed to detection, of human face images [85–88]. We note that the present results reflect successful decoding of responses to specific image exemplars, and not to individuals *per se*. Decoding of identity would require that a classifier discriminate responses to images of a given individual over variations of pose and lighting. The same is true to a substantial degree for the decoding of other images and image categories in the stimulus set used here and in previous studies.

**Fig 12:**
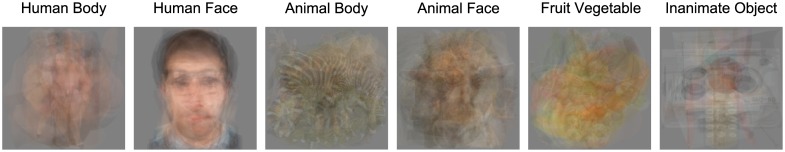
Stimulus set, averaged by category.

The images in this stimulus set differ substantially in low-level feature content. Analysis of computational and neural representations of the 92-image set from which the current stimulus set was derived reveals that Human Face images are well differentiated from those of other categories on the basis of silhouette and luminance characteristics [42]. As can be appreciated from an inspection of the category means of the present stimulus set (Fig 12), the Human Face stimuli in particular share low-level image properties, which preserve a prototypically discernable Human Face after averaging. These stimulus similarities likely contribute to the coherence and distinctness of this category in the representational space of the present study [89].

Category-specific effects at the P1 latency disappear when power spectral differences across categories are eliminated [21], so one might expect that the early-latency differences we observe would be smaller if these features were eliminated. Beyond this, as noted above, it is not entirely clear to what extent image similarity, rather than abstract semantic category membership, drives classification with the current image set. The present analysis shows that a broadly similar representational space exists across human BOLD, MEG, and EEG measures, as well as for macaque IT single units. Nonetheless, it is still the case that in natural viewing of object categories, low-level feature differences are present and provide information for object category processing, as has been recently shown in human fMRI and monkey IT studies [90, 91]. It would be useful to determine how categorical representations would change and what their temporal evolution would be if low-level features were controlled and if classifiers were applied to the problem of invariant object recognition.
